# Preferences of first-degree relatives of gastric cancer patients for gastric cancer screening: a discrete choice experiment

**DOI:** 10.1186/s12885-021-08677-9

**Published:** 2021-08-26

**Authors:** Hui-qin Li, Hui Xue, Hua Yuan, Guang-ying Wan, Xiu-ying Zhang

**Affiliations:** 1 Department of Fundamental Nursing, School of Nursing, Jilin University, 965 Xinjiang Street, Changchun, 130021 Jilin Province P. R. China; 2 Department of Histology & Embryology, College of Basic Medical Sciences, Jilin University, 126 Xinmin Street, Changchun, 130021 Jilin Province P. R. China

**Keywords:** Gastric cancer, Cancer screening, Discrete choice experiment, Preferences

## Abstract

**Background:**

It is very necessary to implement gastric cancer screening in China to reduce the mortality of gastric cancer, but there are no national screening guidelines and programs. Understanding of individual preferences is conducive to formulating more acceptable screening strategies, and discrete choice experiments can quantify individual preferences. In addition, the first-degree relatives of gastric cancer patients are at high risk for gastric cancer. Compared with those without a family history of gastric cancer, the risk of gastric cancer in first-degree relatives of gastric cancer patients is increased by 60%. Therefore, a discrete choice experiment was carried out to quantitatively analyse the preferences of first-degree relatives of gastric cancer patients for gastric cancer screening to serve as a reference for the development of gastric cancer screening strategies.

**Methods:**

A questionnaire was designed based on a discrete choice experiment, and 342 first-degree relatives of gastric cancer patients were investigated. In STATA 15.0 software, the data were statistically analysed using a mixed logit model.

**Results:**

The five attributes included in our study had a significant influence on the preferences of first-degree relatives of gastric cancer patients for gastric cancer screening (*P* < 0.05). Participants most preferred the sensitivity of the screening program to be 95% (coefficient = 1.424, *P* < 0.01) with a willingness to pay 2501.902 Yuan (95% CI, 738.074–4265.729). In addition, the participants’ sex and screening experiences affected their preferences. An increase in sensitivity 35 to 95% had the greatest impact on the participants’ willingness to choose a gastric cancer screening program.

**Conclusion:**

The formulation of gastric cancer screening strategies should be rooted in people’s preferences. The influence of sex differences and screening experiences on the preferences of people undergoing screening should be considered, and screening strategies should be formulated according to local conditions to help them play a greater role.

**Supplementary Information:**

The online version contains supplementary material available at 10.1186/s12885-021-08677-9.

## Background

Gastric cancer, the third most common cause of cancer death in the world, according to global cancer statistics released by the WHO in February 2021, in 2020, the number of deaths from gastric cancer worldwide reached 768,793, while the number of deaths from gastric cancer in China reached 373,789, accounting for approximately 48.6% of global deaths from gastric cancer [https://gco.iarc.fr/today/home]. At present, approximately 90% of gastric cancer cases in China are found in advanced stages. Even when patients with advanced gastric cancer receive surgical treatment, the 5-year survival rate is still less than 30% [1]. Sumiyama’s research showed that the 5-year survival rate of patients with early gastric cancer can exceed 90% after treatment and that such patients can even achieve remission [2]. However, the diagnosis and treatment rate of early gastric cancer in China is less than 10%, which is much lower than that in Japan and South Korea (70%) [3–5]. The implementation of gastric cancer screening to reduce the gastric cancer incidence and mortality in China is a public health issue that urgently needs to be resolved, but no national screening guidelines or programmes are in place [6]. “Three-year Action Plan for Cancer Prevention and Control in China (2015-2017)” clearly states that cancer screening and early diagnosis and treatment strategies in China need to be promoted and improved [7].

Focusing on the preferences of participants in formulating screening strategies will help increase participants’ acceptance of screening strategies, increase participation rates, and enable screening strategies to play a greater role. However, there has been no research on preferences for gastric cancer screening. A discrete choice experiment (DCE) is a method to elicit individual preferences. It is based on the premise that goods, services or policies can be described from their corresponding attributes (or characteristics) and that individual preferences for them depend on the levels of these attributes [8, 9]. Therefore, in a DCE, participants are provided with hypothetical scenarios composed of attributes and levels and are asked to choose among these scenarios. Then, participants’ preferences are explored based on the results of their trade-off of attributes and levels [10]. DCEs have often been used in the health field to evaluate the intensity of preferences and the value of interventions in various health policy contexts [10–12].

China is the largest developing country in the world, with a total population of 1.39538 billion in 2018, and the country’s population will stabilize in the next 20 years [13]. Screening the entire population is very difficult and requires considerable financial expenditure. Studies have confirmed that the incidence of precancerous lesions in first-degree relatives (FDRs) of gastric cancer patients is higher than that in natural populations [14–16]. In addition, compared to people without a family history of gastric cancer, FDRs of gastric cancer patients have a 60% increased risk of developing gastric cancer [17] and are 1.56 times more likely to be diagnosed with the disease [18]. Japan and South Korea have set 40 as the starting age for gastric cancer screening [19]. The incidence of gastric cancer in people over 40 in China has increased significantly, and some experts have suggested that this age should be set as the starting age for gastric cancer screening [20]. Therefore, the purpose of this study was to use a DCE to explore the preferences of FDRs of gastric cancer patients over 40 years of age for gastric cancer screening to provide references for the establishment of a gastric cancer screening system in China and in other developing countries.

## Method

### Aim

The aim of this study was to use a DCE to explore the preferences of FDRs of gastric cancer patients over 40 years of age for gastric cancer screening to provide references for the establishment of a gastric cancer screening system in China and other developing countries.

### Design

A DCE was used as the research design for this study. The DCE design process mainly involved the following two stages:

1. Determination of attributes and levels;.

2. Choice set generation and questionnaire design.

### Determination of attributes and levels

Attributes and their levels were identified based on literature reviews, in-depth interviews, a focus group discussion, and expert consultations. First, literature retrieval was conducted using electronic databases such as PubMed, Web of Science, China National Knowledge Infrastructure (CNKI) and WANFANG DATA. Literatures on the following topics were examined: people’s views and attitudes towards gastric cancer screening; factors affecting people’s participation in gastric cancer screening; the current state of gastric cancer screening; and the management of FDRs of gastric cancer patients. In-depth interviews were conducted with the FDRs of 13 gastric cancer patients to identify more attributes and possible levels. In addition, a focus group discussion was held with 11 FDRs of gastric cancer patients. In the focus group, the participants were provided with a list of attributes identified from our literature review and in-depth interviews, and they were asked to vote on the importance of the attributes. The participants were then asked to discuss the levels and descriptions of the five attributes with the most votes until they reached a consensus. Health economists and clinical experts were then consulted to determine the final attributes and levels. See Table 1 for further details on the attributes and levels.

**Table 1:** Attributes and levels

Attributes	Description	Levels	Description
Cost(¥)	Out-of-pocket costs for participating in each screening; these not include transport, time off from work, and carer costs.	200 CNY	Each screening needs to pay 200 yuan RMB
		400 CNY	Each screening needs to pay 400 yuan RMB
		600 CNY	Each screening needs to pay 600 yuan RMB
Waiting time, hour	The time of waiting in line to participate in the screening after arriving at the screening institution.	1 h	The time to wait in line for screening is 1 h.
		3 h	The time to wait in line for screening is 3 h.
		5 h	The time to wait in line for screening is 5 h.
Pain	Is there pain or discomfort?	Severe	The pain is obvious and severe, which may be unbearable for you.
		Mild	You may feel pain or discomfort.
		None	You feel no pain or discomfort
Frequency	How often will the screening test be done?	Once a year	You will have the screening test every years.
		Once every two years	You will have the screening test every 2 years.
		Once every three years	You will have the screening test every 3 years.
Sensitivity	Is it accurate if you DO have cancer?	35%	If you DO have cancer, the test will miss it 65 out of 100 times
		65%	If you DO have cancer, the test will miss it 35 out of 100 times
		95%	If you DO have cancer, the test will miss it 5 out of 100 times

### Choice set generation and questionnaire design

**Fig. 1:**
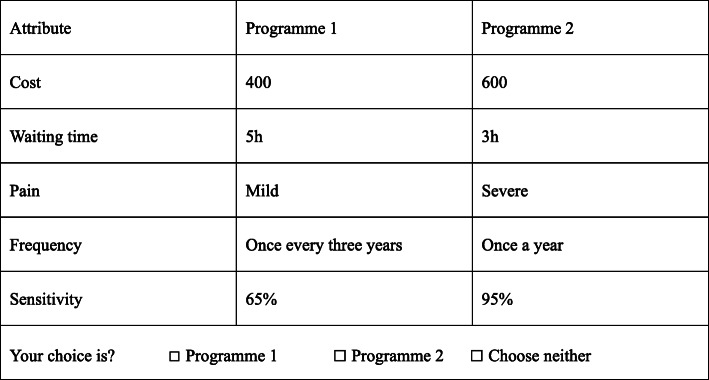
An example of choice set: which of these programmes would you prefer?

This study contains 5 attributes, each of which has 3 levels. Ngene 1.2 is a software program used to generate experimental designs for choice experiments, which can realize an efficient design and divide the design into blocks according to the principle of least correlation (www.choice-metrics.com). Therefore, in Ngene software, an efficient design was used to generate 36 choice sets. The pwcorr command in Stata 15.0 was used to estimate the correlations of the levels of each attribute, and all of the correlations were found to be low enough to not cause concern (the results of correlation estimations were given in Supplement 1). The level balance of the design applied in this study is provided in Supplement 2, which shows that the design achieved a good, if not perfect, level balance. To further reduce the cognitive burden on the respondents, the 36 choice sets were randomly divided into 4 blocks (the results of correlation estimations are shown in Supplement 1). Each version includes a repeated choice set to test the consistency of the respondents’ choices. Respondents randomly accepted one of the four versions, and each version contained 10 choice sets (For the final choice sets, please see the Supplement 3). An example of a choice set is shown in Fig. 1.

At the beginning of the questionnaire was an introduction to the research background, the content of the questionnaire and the requirements for completing the questionnaire. The first part of the questionnaire was a general information questionnaire focused on sociodemographic characteristics such as sex, age, income, and the screening experiences of the participants. The second part of the questionnaire included the DCE survey. An example choice set was given at the beginning of this section. The respondents were asked to complete 10 choice sets. Each choice set contained 2 alternatives and 1 exit item, and the respondents were invited to choose their favourite option in each choice set.

A pilot test was conducted on 20 FDRs of gastric cancer patients to estimate face validity and identify any problems in understanding the requirements of the questionnaire or of individual questions. Based on feedback received from the pilot test, the wording of the questionnaire was revised to improve clarity.

### Research objects and ethics

The objects of this study are FDRs of gastric cancer patients. As inclusion criteria, we applied the following: 1. parents, siblings and children of gastric cancer patients; and 2. age ≥ 40 years; and 3. the ability to read and understand text. As exclusion criteria, we applied the following: 1. those with a history of gastric cancer or who have been diagnosed with gastric cancer; and 2. those unwilling to participate in this research. This study was approved by the Ethics Committee of the School of Nursing of Jilin University.

### Data collection

We randomly included individuals who met the inclusion and exclusion criteria in the Sichuan, Jilin, Gansu, Beijing and Hainan provinces of China. Mainly hard-copy questionnaires were used for data collection. For participants who preferred an electronic version of the questionnaire, electronic questionnaires were provided through the WeChat platform or email. The versions of the questionnaire were randomly distributed, and the same number of each version was distributed.

### Data analysis

The data were double-entered into EpiData 3.1 software and transferred to Stata 15.0 software for processing and analysis. Descriptive statistics on the participants’ sociodemographic characteristics were recorded. A mixed logit model (also known as a random parameter logit) accounting for the potential preference heterogeneity of the participants was used to analyse the preferences of FDRs of gastric cancer patients for gastric cancer screening. It is assumed that all coefficients of attribute levels are random with a normal distribution and are freely correlated. Cost was specified as a continuous variable in the model to facilitate the calculation of the willingness to pay (WTP), that is, the relative monetary value that FDRs of gastric cancer patients place on various aspects of screening programmes. The remaining attributes were designated as dummy variables. We also conducted a simulation study to understand the extent to which the probability of choosing a given screening programme changes as the attribute level changes.

The incidence of gastric cancer in men is 1.8–2 times that in women [21], while in China, the incidence of gastric cancer in men is 2.1 times that in women [22]. In addition, most people participate in gastric cancer screening only after exhibiting abnormalities [23]. Screening experience may indicate whether the body shows abnormalities and the degree of concern for gastric cancer. Therefore, people with screening experience and those without screening experience may have different preferences for gastric cancer screening. Therefore, we conducted subgroup analyses of the participants by sex and screening experience to explore the impact of these factors on gastric cancer screening preferences of FDRs of gastric cancer patients.

## Results

### Participants’ characteristics

A total of 400 FDRs of gastric cancer patients accessed the survey, of whom 27 did not complete the questionnaire and 31 of whom failed the consistency test and were excluded. Finally, 342 FDRs of gastric cancer patients (85.5%) were included in the analysis.

Among the 342 respondents, the majority (54.68%) were women, and 58.77% had a junior high school education. The largest portion of respondents (49.12%) were 50–59 years old. A total of 49.42% of the respondents had a monthly income of 2000–6000 yuan, and 47.95% of the respondents lived in a city. In terms of screening experience, more than half (56.73%) of the respondents had no screening experience. A total of 52.34% of the respondents had no history of smoking, and 57.89% of the respondents had a history of drinking. The participants’ specific sociodemographic characteristics are shown in Table 2.

**Table 2:** Demographic characteristics of respondents

Characteristics	Respondents (*N* = 342)	
	N	%
Sex		
Male	155	45.32
Female	187	54.68
Age, years		
40–49	113	33.04
50–59	168	49.12
60–69	61	17.84
Highest level of education		
Primary school and below	16	4.68
Junior high school	201	58.77
Senior high school	68	19.88
College degree and above	57	16.67
Screening experience		
Yes	148	43.27
No	194	56.73
Monthly income		
<2000	26	7.60
2000–6000	169	49.42
6000–10,000	88	25.73
≥ 10,000	59	17.25
Location		
City	164	47.95
Town	76	22.22
Country	102	29.83
Smoking history		
Yes	163	47.66
No	179	52.34
History of drinking		
Yes	198	57.89
No	144	42.11

### Overall results

The mixed logit estimates for the total sample and the calculated results for the willingness to pay are reported in Table 3.

**Table 3:** Mixed logit results and WTP

Attribute levels (reference level)	coefficient (s.e)	SD(s.e)	WTP (CNY)	95% CI	
Cost Waiting time (5 h)	−0.001^**^(0.001)	0.000 (0.000)	–	–	–
3 h	0.292^*^(0.115)	0.460^**^(0.174)	513.443	−15.198	1042.084
1 h Pain (Severe)	0.542^**^(0.154)	0.820^**^(0.233)	953.082	159.389	1746.775
Mild	0.707^**^(0.132)	0.951^**^(0.122)	1241.728	189.703	2293.753
No Frequency (Once every three years)	1.340^**^(0.205)	1.990**(0.207)	2353.713	572.221	4135.205
Once every two years	0.377^**^(0.114)	0.327^*^(0.165)	663.266	12.771	1313.760
Once a year Sensitivity (35%)	0.638^**^(0.128)	0.658^**^(0.226)	1120.583	170.703	2070.463
65%	0.897^**^(0.123)	0.885^**^(0.159)	1575.725	395.888	2755.563
95%	1.424^**^(0.157)	1.477^**^(0.199)	2501.902	738.074	4265.729
Log likelihood	−2013.711				
Number of obs	9234				
N	342				

*p < 0.05,**p < 0.01

The signs for all of the attributes are as expected, and all of the attributes have a significant influence on preferences for gastric cancer screening. The signs for all attribute levels are positive, except that the sign for the attribute “cost” is negative. The FDRs of gastric cancer patients valued sensitivity of 95% most (coefficient = 1.424, *P* < 0.01), followed by no pain (coefficient = 1.340, *P <* 0.01). Although cost has a significant impact on the preferences of FDRs of gastric cancer patients for gastric cancer screening (coefficient = − 0.001, *P <* 0.01), it was not ranked as important as other attribute levels. The standard deviations of all attribute levels are significant (*P* < 0.05), confirming the existence of preference heterogeneity.

The WTP measures how much respondents are willing to pay to improve the features of other screening programmes or how much compensation they need to receive to accept the undesired features of screening programmes. The WTP values for sensitivity and pain clearly demonstrate the importance of these two attributes. Respondents were willing to pay 2501.902 CNY (95% CI, 738.074–4265.729) for sensitivity of 95% and 2353.713 CNY (95%CI, 572.221–4135.205) for no pain. The WTP for waiting time of 3 h for each screening was found to be the lowest at 513.443 CNY (95% CI, − 15.198-1042.084).

### Results of subgroup analyses

The results of a subgroup analysis conducted by sex are presented in Table 4.

**Table 4:** Results of subgroup analysis based on sex

Attribute levels (reference level)	Male					Female				
	coefficient (s.e)	SD(s.e)	WTP (CNY)	95% CI		coefficient (s.e)	SD(s.e)	WTP (CNY)	95% CI	
Cost	−0.0006534^*^ (0.0002826)	0.000^**^(0.000)	–	–	–	−0.002^**^(0.000)	0.000^**^(0.000)	–	–	–
Waiting time (5 h)										
3 h	0.346^*^(0.149)	0.504^*^(0.201)	529.043	−205.458	1263.545	0.535^**^(0.126)	0.321 (0.207)	253.751	143.354	364.147
1 h	1.005^**^(0.247)	1.369^**^(0.297)	1537.903	74.409	3001.397	0.762^**(^0.161)	0.587^*^(0.262)	361.501	181.124	541.879
Pain (Severe)										
Mild	0.608^**^(0.148)	0.118 (0.308)	929.791	−76.306	1935.890	0.611^**^(0.169)	0.319 (0.284)	289.833	126.950	452.716
No	0.743^**^(0.233)	1.480^**^(0.252)	1137.867	−66.606	2342.279	1.797^**^ (0.273)	1.587^**^(0.272)	852.135	512.386	1191.885
Frequency (Once every three years)										
Once every two years	0.547^**^ (0.154)	0.125 (0.049)	836.906	−76.345	1750.158	0.384^*^(0.178)	0.708^**^(0.218)	182.299	17.751	346.846
Once a year	0.957^**^(0.176)	0.629^*^(0.295)	1465.312	54.404	2876.220	1.198^**^(0.212)	1.058^**^(0.236)	567.935	302.961	832.910
Sensitivity (35%)										
65%	0.661^**^ (0.170)	0.636^**^ (0.214)	1011.652	6.462	2016.842	0.233 (0.163)	0.479^**^(0.183)	110.634	−54.142	275.411
95%	1.064^**^(0.221)	0.978^**^ (0.317)	1628.547	85.857	3171.238	1.166^**^(0.229)	1.353^**^(0.265)	552.799	277.145	828.453
log likelihood	− 914.59772					− 1082.0553				
Number of obs	4185					5049				
N	155					187				

*p < 0.05, **p < 0.01

Sensitivity of 95% was found to be the attribute level most valued by the male respondents (coefficient = 1.064, *p* < 0.01), consistent with the overall results, and these respondents were willing to pay 1628.547 CNY to obtain this attribute level. The second most important attribute level was found to be waiting time for each screening of 1 h (coefficient = 1.005, *p* < 0.01), and male respondents were willing to pay 1537.903 CNY to obtain this attribute level. Different from the overall results and the results of the male respondents, female respondents selected an absence of pain as the most important attribute level (coefficient = 1.797, *p <* 0.01), followed by screening for gastric cancer once a year (coefficient = 1.198, *p <* 0.01); these participants were willing to pay 852.135 CNY and 567.935 CNY for these features, respectively.

The results of a subgroup analysis conducted by experience with gastric cancer screening are presented in Table 5. The results show that, consistent with the overall results, 95% test sensitivity (coefficient = 1.729, *P* < 0.01) was most valued by the respondents with screening experience, and they were willing to pay 2063.477 CNY for this attribute level. The second most important attribute level for respondents with screening experience was identified as screening once a year (coefficient = 0.998, *p* < 0.01), and these participants were willing to pay 1191.155 CNY for this attribute level. Interestingly, respondents with screening experience showed a greater preference for mild pain than for no pain. In contrast, the attributes “pain” and “waiting time” had a greater impact on the preferences of respondents without screening experience. The most important attribute level for respondents without screening experience was found to be no pain (coefficient = 1.376, *p* < 0.01), followed by waiting time of 1 h (coefficient = 1.207, *p <* 0.01), and these individuals were willing to pay 1345.418 CNY and 1180.131 CNY for these attribute levels, respectively.

**Table 5:** Results of subgroup analysis based on screening experience

Attribute levels (reference level)	No screening experience					Having screening experience				
	coefficient (s.e)	SD(s.e)	WTP (CNY)	95% CI		coefficient (s.e)	SD(s.e)	WTP (CNY)	95% CI	
Cost	−0.001**(0.000)	0.000**(0.000)	–	–	–	−0.0008381** (0.0002691)	0.000 (0.000)			
Waiting time (5 h)										
3 h	0.381**(0.134)	0.745**(0.183)	372.790	47.379	698.201	0.369**(0.142)	0.335 (0.256)	440.650	−4.460	885.761
1 h	1.207** (0.162)	0.712 (0.169)	1180.131	555.074	1805.188	0.369**(0.142)	0.335 (0.256)	440.650	−4.460	885.761
Pain (Severe)										
Mild	0.514**(0.123)	0.201**(0.187)	502.265	189.266	815.264	0.558* (0.228)	1.333**(0.233)	665.871	−50.622	1382.364
None	1.376** (0.132)	1.177**(0.193)	1345.418	718.431	1972.403	0.432**(0.148)	0.145 (0.372)	514.952	30.124	999.780
Frequency (Once every three years)										
Once every two years	0.596**(0.121)	0.116 (0.189)	582.855	297.278	868.432	0.455**(0.165)	0.373 (0.261)	542.954	4.489	1081.419
Once a year	0.926**(0.115)	0.066 (0.318)	905.421	473.413	1337.429	0.998**(0.229)	1.271**(0.291)	1191.155	317.243	2065.067
Sensitivity (35%)										
65%	0.335*(0.135)	0.719**(0.160)	327.400	93.240	561.561	0.646**(0.157)	0.560*(0.249)	770.735	134.700	1406.769
95%	0.887**(0.158)	1.087**(0.168)	867.541	582.851	1302.231	1.729**(0.247)	1.673**(0.272)	2063.477	587.478	3539.477
log likelihood	− 1432.2078					−926.18352				
Number of obs	5238					3996				
N	194					148				

*p < 0.05.**p < 0.01

### Simulated screening programme preferences with changes in programme characteristics

**Fig. 2:**
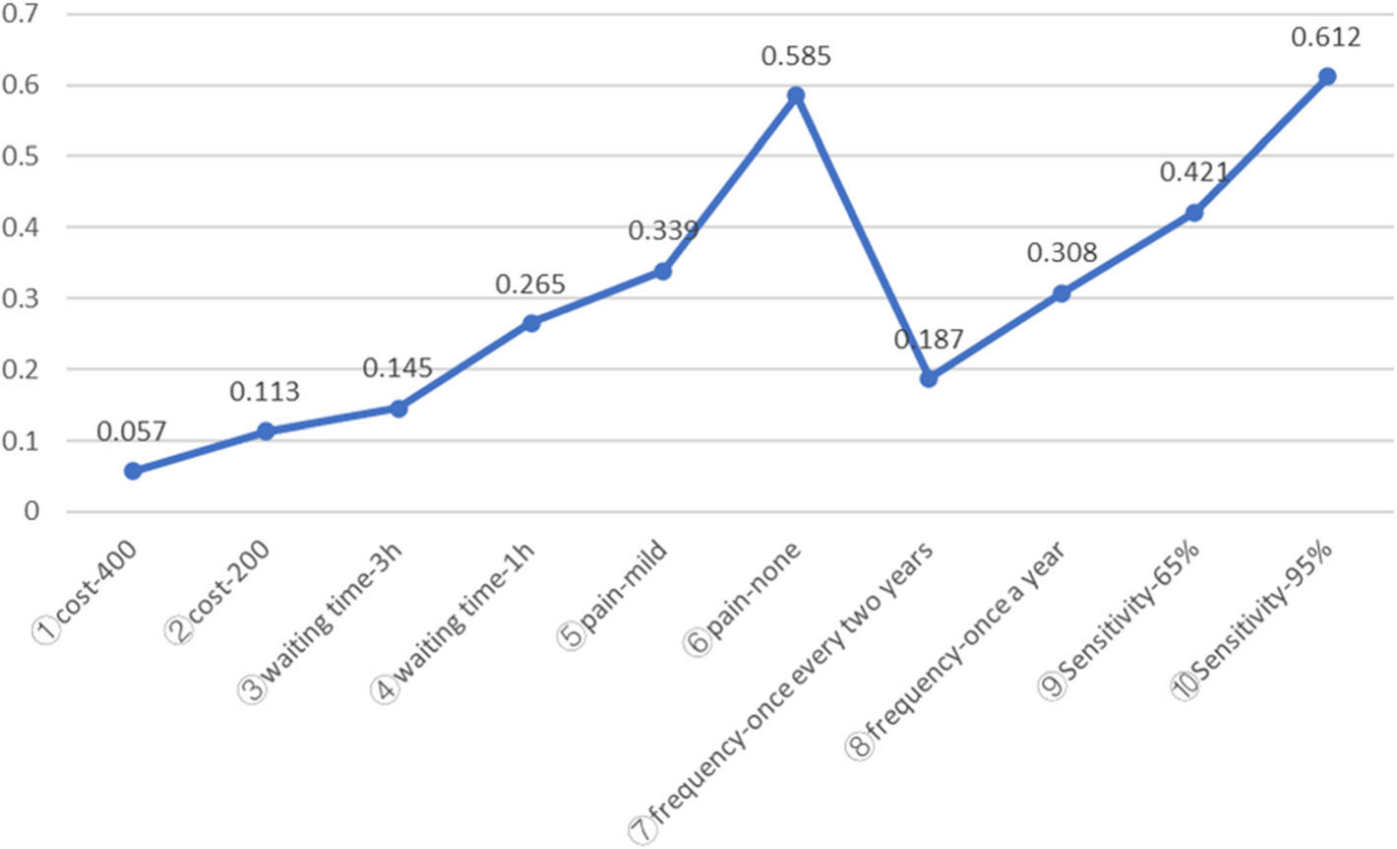
Simulated screening programme preferences with changes in programme characteristics. Baseline screening programme: Cost-600, Waiting time-5 h, Pain-severe, Frequency-once every three years, Sensitivity-35%

The possibilities of accepting a baseline screening programme after a change in the level of one of the screening programme attributes were simulated, and the main results are reported in Fig. 2.

An increase in sensitivity from 35 to 95% had the greatest impact on the respondents’ preferences, followed by a change in pain levels from severe pain to no pain, and the probability of screening programme selection increased by 0.612 and 0.585, respectively. In comparison, when the cost changed from 600 to 400 and from 600 to 200, the probability of screening programme selection increased by 0.057 and 0.113, respectively, which are less than the increase caused by changes in the levels of other attributes.

## Discussion

To our knowledge, this work presents the first DCE examining the preferences of FDRs of gastric cancer patients for gastric cancer screening and the screening programme-related characteristics that could motivate them to accept one programme over another. We found sensitivity and pain to be highly valued by FDRs of gastric cancer patients, who were willing to pay more for higher sensitivity and less pain. In addition, the sex and screening experiences of FDRs of gastric cancer patients affected their preferences for gastric cancer screening. Although changes in sensitivity had the greatest impact on the selection of gastric cancer screening programmes, pain reduction and changes to the frequency of screening also had a greater impact on the selection of gastric cancer screening programmes.

Sensitivity reflects the accuracy of screening methods. The FDRS of gastric cancer patients indicated the most important attribute level as a test sensitivity of 95%, and they were willing to pay the most for this feature, which seems to show that such individuals value the validity of testing. A previous study found FDRs of gastric cancer patients to report a self-perceived absolute lifetime risk of developing gastric cancer of 54% and 68% of FDRs of gastric cancer patients to show concerns of developing cancer [24]. This concern may demonstrate why such individuals emphasize the validity of gastric cancer screening.

Pain levels had drew considerable attention from FDRs of gastric cancer patients, as they were willing to pay more to experience less pain. We hypothesized the participants’ emphasis on “pain” to mainly be caused by a fear of pain from gastroscopy. China has a large population and a relative lack of medical and health resources, and opportunistic gastroscopy for asymptomatic individuals is currently the main means of gastric cancer screening used in the country [25, 26]. Qi Liu [27] found a fear of undergoing gastroscopy to be a main reason for not accepting gastric cancer screening. Furthermore, when asked about the most acceptable gastric cancer screening method, 60.2% of the participants preferred blood testing, only 29.8% preferred endoscopy [27], and pain was identified as the main disadvantage of endoscopy [28]. These results illustrate the importance that participants attach to pain levels and confirm our hypothesis.

The sex and screening experiences of FDRs of gastric cancer patients were found to affect their preferences for gastric cancer screening. The incidence of gastric cancer is higher in men than in women [21, 22]. In addition, men have more negative views of cancer than women [29], and the need for early detection and prevention of cancer is higher for men than for women [30]. It comes to cancer, men reminded of death for cancer in the first, while women reminded of fear, terror, suffering agony or pain from the disease [30], and Han’s research indicated that women focus on pain more than men [17]. This explained why our male participants valued higher sensitivity the most while our female respondents prioritised experiencing less pain the most. One study reported that most people are screened for gastric cancer only after they have symptoms [23] and found an asymptomatic response to be the most common self-reported reason for not screening for gastric cancer [27, 31]. In China, endoscopy for opportunistic screening is the main method of gastric cancer screening [25, 32], while a fear of endoscopy is one of the main reasons why people do not accept gastric cancer screening [27, 31]. This may explain why our participants with screening experience valued higher sensitivity, while participants without screening experience prioritised less pain. It should be noted that the respondents with screening experience preferred mild pain more over no pain. People at high risk for gastric cancer believe that all effective screening methods will cause pain [33]. In China, individuals with gastric cancer screening experience are mostly high-risk groups or people with physical symptoms. Furthermore, one study found that people with screening experience prefer blood testing, which causes only mild pain [27]. This may explain why the coefficient for mild pain is larger than that for no pain for the group with screening experience. In the future, qualitative researches can be carried out to more deeply explore FDRs of gastric cancer patients’ views on the pain caused by screening methods.

The simulation results indicate the extent to which changes in specific attribute levels and/or attribute combinations affect the selection of screening programmes. Increasing sensitivity from 35 to 95% had the greatest impact on the selection of screening programmes, followed by a change in pain levels from severe to none. The sensitivity of endoscopy to gastric cancer is 88.5–97.7% [34, 35], and mortality from gastric cancer can be reduced by 47% after endoscopic screening [36]. If endoscopy were used to screen everyone to achieve higher sensitivity, this could affect people’s acceptance of screening programmes. The influence of changing pain levels from "severe" to "no" on the selection of screening programmes was second only to increased sensitivity, but pain caused by endoscopy is severe. The use of gastrointestinal ultrasound is not restricted by age, cardiopulmonary function or implants in the body, and its advanced gastric cancer detection rate is 90% [37]. However, because this method is affected by patient obesity and flatulence, the detection of micro tumours is greatly influenced by the experience and manipulation of professionals who perform the examination [38, 39]. Therefore, it may not be feasible to use this method for population-based screening in China. A combined use of the *H. pylori* antibody and serum pepsinogen test has been rapidly disseminated, and the introduction of this combined method as population-based screening approach has been greatly anticipated [40]. Twice as many people view blood tests as the most acceptable screening method as those who prefer endoscopy [27]. Mi-Mi Liu and his colleagues established three data mining models adopting a noninvasive and painless method that can effectively assess the risk of developing early gastric cancer [41]. From the results of our study, it may be more feasible and effective to use data mining models or serological testing to conduct preliminary screening for FDRs of gastric cancer patients and then perform further endoscopy and pathological biopsies on the selected high-risk populations because this hierarchical screening strategy is less painful and costly.

The self-perceived absolute lifetime risk of developing gastric cancer in FDRs of gastric cancer patients was recorded as 54%, and 84% of the FDRs of gastric cancer patients realized that the earlier gastric cancer is detected, the higher the cure rate is [24]. This high self-perceived absolute lifetime risk of gastric cancer and understanding of the early diagnosis and treatment of gastric cancer may lead such individuals to assume that the interval between screenings is short, which may lead to a change in screening frequency from once every 3 years to once a year also having a great influence on the selection of screening programmes. The literatures on the optimal frequency of gastric cancer screening are limited. In South Korea and Japan, gastric cancer screening is conducted every 2 years [42, 43]. Chinese experts have recommended a certain frequency of gastric cancer screening with endoscopy [20], but people’s satisfaction with frequency and acceptance of this frequency are not yet known, and no recommendations have been made on the frequency of screening with serological methods. According to our results, gastric cancer screening for FDRs of gastric cancer patients once a year may improve their participation in and satisfaction with gastric cancer screening. In addition, including cancer screening under medical insurance coverage and increasing reimbursement rates can promote participation in gastric cancer screening. However, as China is a developing country with a large population, cancer screening cannot be included in medical insurance in the near future, as this will require considerable financial support. Fortunately, reducing the waiting time for screening can also facilitate the use of screening options. From the results of our research and the current situation in China, we put forward the following suggestions:

1. Data mining models can be used to conduct a preliminary screening once a year for female FDRs of gastric cancer patients and for FDRs of gastric cancer patients without screening experience, and then further endoscopic examination and pathological biopsy can be performed for the selected high-risk population.
2. For FDRs of gastric cancer patients with screening experience, gastric cancer screening can be carried out once a year with endoscopy.
3. Set up more gastric cancer screening institutions to evacuate the screening population, and develop applications to allow people undergoing gastric cancer screening to make appointments and understand the precautions for gastric cancer screening on the application to reduce the waiting time for screening.

Our sample of participants was collected from five provinces in China, reducing bias from sampling and improving the objectivity of our results. Our study also presents some limitations. First, similar to other DCEs, our study explored the stated preferences of the FDRs of gastric cancer patients for gastric cancer screening, and we cannot verify the degree of consistency between stated preferences and revealed preferences. Second, the preferences of FDRs of gastric cancer patients for gastric cancer screening were assessed under the Chinese gastric cancer screening system, which limits the generalizability of our results to other countries. However, the results of our study have valuable implications for improving gastric cancer screening systems in China and other developing countries.

## Conclusion

The formulation of gastric cancer screening strategies should be rooted in people’s preferences. The influence of sex differences and screening experience on the preferences of people undergoing screening should be considered, and screening strategies should be formulated according to local conditions to help them play a more influential role.

## Supplementary Information

**Additional file 1: Supplement 1**. The results of estimate the correlations in the levels of each attribute.

**Additional file 2: Supplement 2**. Level balance of the design in our study.

**Additional file 3: Supplement 3**. Final choice sets.

## Data Availability

The data and materials used to support the findings of this study are included within the article, and all data included in this study are available upon request by contact with the corresponding authors.

## Abbreviations

DCE: Discrete choice experiment
FDRs: First-degree relatives
WTP: Willingness to pay
