# Supplementary material for: Preferences of first-degree relatives of gastric cancer patients for gastric cancer screening: a discrete choice experiment
Source: BMC Cancer. 2021 Aug 26;21:959. doi: 10.1186/s12885-021-08677-9 (PMC8393792; doi:10.1186/s12885-021-08677-9)
Supplement: Supplementary file 1 — Additional file 1: Supplement 1. The results of estimate the correlations in the levels of each attribute. [file 12885_2021_8677_MOESM1_ESM.docx]

**Supplement 1. The results of estimate the correlations in the levels of each attribute**

**36 choice set**

|  | Cost | time-1h | time-3h | time-5h | pain-none | pain-mild | pain-severe | frequency-1 | frequency-2 | frequency-3 | sensitivity-35% | sensitivity-65% | sensitivity-95% |
| --- | --- | --- | --- | --- | --- | --- | --- | --- | --- | --- | --- | --- | --- |
| Cost | 1.0000 |  |  |  |  |  |  |  |  |  |  |  |  |
| time-1h | 0.0000 | 1.0000 |  |  |  |  |  |  |  |  |  |  |  |
| time-3h | 0.0000 | -0.5000* | 1.0000 |  |  |  |  |  |  |  |  |  |  |
| time-5h | 0.0000 | -0.5000* | -0.5000* | 1.0000 |  |  |  |  |  |  |  |  |  |
| pain-none | -0.0365 | 0.0211 | -0.0421 | 0.0211 | 1.0000 |  |  |  |  |  |  |  |  |
| pain-mild | 0.0357 | -0.0206 | 0.0413 | 0.0206 | -0.4997* | 1.0000 |  |  |  |  |  |  |  |
| pain-severe | 0.0000 | 0.0000 | 0.0000 | 0.0000 | -0.4845* | -0.5157* | 1.0000 |  |  |  |  |  |  |
| frequency-1 | 0.0000 | 0.0000 | -0.0000 | 0.0000 | 0.0211 | -0.0206 | 0.0000 | 1.0000 |  |  |  |  |  |
| frequency-2 | 0.0000 | 0.0000 | 0.0000 | -0.0000 | 0.0211 | -0.0206 | 0.0000 | -0.5000* | 1.0000 |  |  |  |  |
| frequency-3 | 0.0000 | -0.0000 | 0.0000 | 0.0000 | -0.0421 | 0.0413 | 0.0000 | -0.5000* | -0.5000 | 1.0000 |  |  |  |
| sensitivity-35% | 0.0000 | 0.0000 | 0.0000 | 0.0000 | 0.0211 | -0.0206 | 0.0000 | -0.0000 | 0.0000 | 0.0000 | 1.0000 |  |  |
| sensitivity-65% | 0.0000 | 0.0000 | 0.0000 | 0.0000 | 0.0211 | -0.0206 | 0.0000 | -0.0000 | 0.0000 | 0.0000 | -0.5000* | 1.0000 |  |
| sensitivity-95% | 0.0000 | 0.0000 | 0.0000 | 0.0000 | 0.0421 | 0.0413 | 0.0000 | 0.0000 | 0.0000 | -0.0000 | -0.5000* | -0.5000* | 1.0000 |

* indicate significance at 5% level respectively.

**Block 1**

|  | Cost | time-1h | time-3h | time-5h | pain-none | pain-mild | pain-severe | frequency-1 | frequency-2 | frequency-3 | Sensitivity -35% | sensitivity-65% | sensitivity-95% |
| --- | --- | --- | --- | --- | --- | --- | --- | --- | --- | --- | --- | --- | --- |
| Cost | 1.0000 |  |  |  |  |  |  |  |  |  |  |  |  |
| time-1h | 0.7071* | 1.0000 |  |  |  |  |  |  |  |  |  |  |  |
| time-3h | -0.4243 | -0.5000* | 1.0000 |  |  |  |  |  |  |  |  |  |  |
| time-5h | -0.2828 | -0.5000* | -0.5000* | 1.0000 |  |  |  |  |  |  |  |  |  |
| pain-none | -0.2828 | -0.2500 | -0.0000 | 0.2500 | 1.0000 |  |  |  |  |  |  |  |  |
| pain-mild | 0.2729 | 0.3508 | -0.4385 | 0.0877 | -0.4385 | 1.0000 |  |  |  |  |  |  |  |
| pain-severe | 0.0228 | -0.0806 | 0.4029 | -0.3223 | -0.56415* | -0.4947* | 1.0000 |  |  |  |  |  |  |
| frequency-1 | 0.2828 | 0.0000 | -0.0000 | 0.0000 | -0.2500 | 0.3508 | -0.0806 | 1.0000 |  |  |  |  |  |
| frequency-2 | 0.2828 | 0.0000 | -0.0000 | -0.0000 | 0.0000 | -0.4385 | 0.4029 | 0.0346 | 1.0000 |  |  |  |  |
| frequency-3 | 0.0000 | -0.0000 | 0.0000 | 0.0000 | 0.2500 | 0.0877 | -0.3223 | -0.5000* | -0.5000* | 1.0000 |  |  |  |
| sensitivity-35% | -0.2507 | 0.0806 | 0.1612 | -0.0806 | -0.5641* | 0.0141 | 0.5325 | -0.0806 | 0.1612 | -0.0806 | 1.0000 |  |  |
| sensitivity-65% | 0.1414 | -0.0000 | 0.2500 | -0.2500 | 0.5000* | -0.4385 | -0.0806 | 0.0000 | -0.2500 | 0.2500 | -0.5641* | 1.0000 |  |
| sensitivity-95% | 0.1240 | 0.0877 | -0.4385 | 0.3508 | 0.0877 | 0.4462 | -0.4947* | 0.0877 | 0.0877 | -0.1754 | -0.4947* | -0.4385 | 1.0000 |

* indicate significance at 5% level respectively.

**Block 2**

|  | Cost | time-1h | time-3h | time-5h | pain-none | pain-mild | pain-severe | frequency-1 | frequency-2 | frequency-3 | Sensitivity -35% | sensitivity-65% | sensitivity-95% |
| --- | --- | --- | --- | --- | --- | --- | --- | --- | --- | --- | --- | --- | --- |
| Cost | 1.0000 |  |  |  |  |  |  |  |  |  |  |  |  |
| time-1h | 0.0000 | 1.0000 |  |  |  |  |  |  |  |  |  |  |  |
| time-3h | -0.0249 | -0.5641* | 1.0000 |  |  |  |  |  |  |  |  |  |  |
| time-5h | 0.0271 | -0.4385 | -0.4947 | 1.0000 |  |  |  |  |  |  |  |  |  |
| pain-none | -0.1353 | 0.0877 | 0.0141 | -0.1077 | 1.0000 |  |  |  |  |  |  |  |  |
| pain-mild | 0.0976 | -0.1581 | 0.2039 | -0.0555 | -0.5547* | 1.0000 |  |  |  |  |  |  |  |
| pain-severe | 0.0271 | 0.0877 | -0.2403 | 0.1692 | -0.3846 | -0.5547* | 1.0000 |  |  |  |  |  |  |
| frequency-1 | 0.3086 | -0.2500 | 0.1612 | 0.0877 | 0.3508 | 0.3953 | 0.0877 | 1.0000 |  |  |  |  |  |
| frequency-2 | 0.0000 | 0.0000 | -0.0806 | 0.0877 | 0.0877 | 0.0791 | -0.1754 | -0.5000* | 1.0000 |  |  |  |  |
| frequency-3 | -0.3086 | 0.2500 | -0.0806 | -0.1754 | -0.4385 | 0.3162 | 0.0877 | -0.5000* | -0.5000* | 1.0000 |  |  |  |
| sensitivity-35% | 0.1895 | 0.0877 | 0.0141 | -0.1077 | -0.3846 | 0.4438 | -0.1077 | -0.4385 | 0.0877 | 0.3805 | 1.0000 |  |  |
| sensitivity-65% | 0.3086 | 0.0000 | -0.0806 | 0.0877 | 0.0877 | -0.3953 | 0.3508 | 0.2500 | -0.0000 | -0.2500 | -0.4385 | 1.0000 |  |
| sensitivity-95% | -0.4725* | -0.0806 | 0.0649 | 0.0141 | 0.2687 | -0.0255 | -0.2403 | 0.1612 | -0.0806 | -0.0806 | -0.4947* | -0.5641* | 1.0000 |

* indicate significance at 5% level respectively.

**Block 3**

|  | Cost | time-1h | time-3h | time-5h | pain-none | pain-mild | pain-severe | frequency-1 | frequency-2 | frequency-3 | Sensitivity -35% | sensitivity-65% | sensitivity-95% |
| --- | --- | --- | --- | --- | --- | --- | --- | --- | --- | --- | --- | --- | --- |
| Cost | 1.0000 |  |  |  |  |  |  |  |  |  |  |  |  |
| time-1h | -0.1396 | 1.0000 |  |  |  |  |  |  |  |  |  |  |  |
| time-3h | 0.3273 | -0.4264 | 1.0000 |  |  |  |  |  |  |  |  |  |  |
| time-5h | -0.1396 | -0.6364* | -0.4264 | 1.0000 |  |  |  |  |  |  |  |  |  |
| pain-none | 0.1443 | -0.0806 | 0.1890 | -0.0806 | 1.0000 |  |  |  |  |  |  |  |  |
| pain-mild | 0.1519 | -0.2403 | -0.0331 | 0.2686 | -0.4385 | 1.0000 |  |  |  |  |  |  |  |
| pain-severe | -0.2791 | 0.2987 | -0.1523 | -0.1688 | -0.5641* | -0.4947* | 1.0000 |  |  |  |  |  |  |
| frequency-1 | -0.1443 | -0.0806 | -0.0945 | 0.1612 | -0.2500 | 0.3508 | -0.0806 | 1.0000 |  |  |  |  |  |
| frequency-2 | 0.0000 | 0.4029 | -0.3780 | -0.0806 | 0.0000 | -0.1754 | 0.1612 | -0.5000* | 1.0000 |  |  |  |  |
| frequency-3 | 0.1443 | -0.3223 | 0.4725* | -0.0806 | 0.2500 | -0.1754 | -0.0806 | -0.5000* | -0.5000* | 1.0000 |  |  |  |
| sensitivity-35% | -0.0000 | -0.2403 | 0.2652 | 0.0141 | 0.0877 | 0.1692 | -0.2403 | 0.3508 | -0.1754 | -0.1754 | 1.0000 |  |  |
| sensitivity-65% | -0.1443 | -0.0806 | -0.3780 | 0.4029 | 0.0000 | -0.1754 | 0.1612 | -0.0000 | 0.0000 | 0.0000 | -0.4385 | 1.0000 |  |
| sensitivity-95% | 0.1396 | 0.2987 | 0.1218 | -0.4026 | -0.0806 | 0.0141 | 0.0649 | -0.3223 | 0.1612 | 0.1612 | -0.4947* | -0.5641* | 1.0000 |

* indicate significance at 5% level respectively.

**Block 4**

|  | Cost | time-1h | time-3h | time-5h | pain-none | pain-mild | pain-severe | frequency-1 | frequency-2 | frequency-3 | Sensitivity -35% | sensitivity-65% | sensitivity-95% |
| --- | --- | --- | --- | --- | --- | --- | --- | --- | --- | --- | --- | --- | --- |
| Cost | 1.0000 |  |  |  |  |  |  |  |  |  |  |  |  |
| time-1h | -6076 | 1.0000 |  |  |  |  |  |  |  |  |  |  |  |
| time-3h | 0.1396 | -0.4947 | 1.0000 |  |  |  |  |  |  |  |  |  |  |
| time-5h | 0.4330 | -0.4385 | -0.5641* | 1.0000 |  |  |  |  |  |  |  |  |  |
| pain-none | 0.1443 | 0.3508 | -0.3223 | 0.0000 | 1.0000 |  |  |  |  |  |  |  |  |
| pain-mild | -0.4187 | 0.0141 | 0.2987 | -3223 | -0.5641* | 1.0000 |  |  |  |  |  |  |  |
| pain-severe | 0.3038 | -0.3846 | 0.0141 | 0.3508 | -0.4385 | -0.4947* | 1.0000 |  |  |  |  |  |  |
| frequency-1 | -0.4330 | 0.3508 | -0.0806 | -0.2500 | 0.2500 | -0.3223 | 0.0877 | 1.0000 |  |  |  |  |  |
| frequency-2 | 0.2887 | -0.4385 | 0.4029 | 0.0000 | 0.0000 | 0.4029 | -0.4385 | -0.5000* | 1.0000 |  |  |  |  |
| frequency-3 | 0.1443 | 0.0877 | -0.3223 | 0.2500 | -0.2500 | -0.0806 | 0.3508 | -0.5000* | -0.5000* | 1.0000 |  |  |  |
| sensitivity-35% | 0.1396 | 0.2686 | -0.4026 | 0.1612 | 0.8864* | -0.6364* | -0.2403 | 0.1612 | -0.0806 | -0.0806 | 1.0000 |  |  |
| sensitivity-65% | -0.2887 | 0.0877 | 0.1612 | -0.2500 | -0.5000* | 0.8864 | -0.4385 | -0.2500 | 0.2500 | 0.0000 | -0.5641* | 1.0000 |  |
| sensitivity-95% | 0.1519 | -0.3846 | 0.2686 | 0.0877 | -0.4385 | -0.2403 | 0.7231* | 0.0877 | -0.1754 | 0.0877 | -0.4947* | -0.4385 | 1.0000 |

* indicate significance at 5% level respectively.
