# Supplementary material for: Preferences of first-degree relatives of gastric cancer patients for gastric cancer screening: a discrete choice experiment
Source: BMC Cancer. 2021 Aug 26;21:959. doi: 10.1186/s12885-021-08677-9 (PMC8393792; doi:10.1186/s12885-021-08677-9)
Supplement: Supplementary file 2 — Additional file 2: Supplement 2. Level balance of the design in our study. [file 12885_2021_8677_MOESM2_ESM.docx]

**Supplement 2. level balance of the design in our study**

| Attribute-level | Number of appearances | % |
| --- | --- | --- |
| Cost-200 | 24 | 33.33 |
| Cost-400 | 24 | 33.33 |
| Cost-600 | 24 | 33.34 |
| Waiting time-1h | 24 | 33.33 |
| Waiting time-3h | 24 | 33.33 |
| Waiting time-5h | 24 | 33.34 |
| Pain-none | 23 | 31.94 |
| Pain-mild | 25 | 34.72 |
| Pain-severe | 24 | 33.34 |
| Frequency- once a year | 24 | 33.33 |
| Frequency-once every two years | 24 | 33.33 |
| Frequency-once every three years | 24 | 33.33 |
| Sensitivity-35% | 24 | 33.33 |
| Sensitivity-65% | 24 | 33.33 |
| Sensitivity-95% | 24 | 33.34 |
