# Supplementary material for: Preferences of first-degree relatives of gastric cancer patients for gastric cancer screening: a discrete choice experiment
Source: BMC Cancer. 2021 Aug 26;21:959. doi: 10.1186/s12885-021-08677-9 (PMC8393792; doi:10.1186/s12885-021-08677-9)
Supplement: Supplementary file 3 — Additional file 3: Supplement 3. Final choice sets. [file 12885_2021_8677_MOESM3_ESM.docx]

**Supplement 3. Final choice sets**

Block 1

1. which of these programmes would you prefer?

| Attribute | Programme 1 | Programme 2 |
| --- | --- | --- |
| Cost | 200 | 400 |
| Waiting time | 5h | 1h |
| Pain | Mild | Severe |
| Frequency | Once a year | Once every two years |
| Sensitivity | 35% | 65% |
| Your choice is? □ Programme 1 □ Programme 2 □ Choose neither | | |

1. which of these programmes would you prefer?

| Attribute | Programme 1 | Programme 2 |
| --- | --- | --- |
| Cost | 600 | 200 |
| Waiting time | 1h | 3h |
| Pain | Mild | Severe |
| Frequency | Once every three years | Once a year |
| Sensitivity | 95% | 35% |
| Your choice is? □ Programme 1 □ Programme 2 □ Choose neither | | |

1. which of these programmes would you prefer?

| Attribute | Programme 1 | Programme 2 |
| --- | --- | --- |
| Cost | 600 | 200 |
| Waiting time | 1h | 3h |
| Pain | Severe | None |
| Frequency | Once every two years | Once every three years |
| Sensitivity | 35% | 65% |
| Your choice is? □ Programme 1 □ Programme 2 □ Choose neither | | |

1. which of these programmes would you prefer?

| Attribute | Programme 1 | Programme 2 |
| --- | --- | --- |
| Cost | 200 | 400 |
| Waiting time | 3h | 5h |
| Pain | None | Mild |
| Frequency | Once every three years | Once a year |
| Sensitivity | 65% | 95% |
| Your choice is? □ Programme 1 □ Programme 2 □ Choose neither | | |

1. which of these programmes would you prefer?

| Attribute | Programme 1 | Programme 2 |
| --- | --- | --- |
| Cost | 200 | 400 |
| Waiting time | 3h | 5h |
| Pain | Severe | None |
| Frequency | Once every two years | Once every three years |
| Sensitivity | 35% | 65% |
| Your choice is? □ Programme 1 □ Programme 2 □ Choose neither | | |

6. which of these programmes would you prefer?

| Attribute | Programme 1 | Programme 2 |
| --- | --- | --- |
| Cost | 600 | 200 |
| Waiting time | 1h | 3h |
| Pain | Mild | Severe |
| Frequency | Once a year | Once every two years |
| Sensitivity | 95% | 35% |
| Your choice is? □ Programme 1 □ Programme 2 □ Choose neither | | |

7. which of these programmes would you prefer?

| Attribute | Programme 1 | Programme 2 |
| --- | --- | --- |
| Cost | 600 | 200 |
| Waiting time | 3h | 5h |
| Pain | Severe | None |
| Frequency | Once a year | Once every two years |
| Sensitivity | 65% | 95% |
| Your choice is? □ Programme 1 □ Programme 2 □ Choose neither | | |

8. which of these programmes would you prefer?

| Attribute | Programme 1 | Programme 2 |
| --- | --- | --- |
| Cost | 200 | 400 |
| Waiting time | 3h | 1h |
| Pain | None | Mild |
| Frequency | Once every two years | Once every three years |
| Sensitivity | 95% | 35% |
| Your choice is? □ Programme 1 □ Programme 2 □ Choose neither | | |

9. which of these programmes would you prefer?

| Attribute | Programme 1 | Programme 2 |
| --- | --- | --- |
| Cost | 400 | 600 |
| Waiting time | 5h | 1h |
| Pain | Severe | None |
| Frequency | Once every three years | Once a year |
| Sensitivity | 35% | 65% |
| Your choice is? □ Programme 1 □ Programme 2 □ Choose neither | | |

10. which of these programmes would you prefer?

| Attribute | Programme 1 | Programme 2 |
| --- | --- | --- |
| Cost | 600 | 200 |
| Waiting time | 1h | 3h |
| Pain | Mild | Severe |
| Frequency | Once every three years | Once a year |
| Sensitivity | 95% | 35% |
| Your choice is? □ Programme 1 □ Programme 2 □ Choose neither | | |

Block 2

1. which of these programmes would you prefer?

| Attribute | Programme 1 | Programme 2 |
| --- | --- | --- |
| Cost | 600 | 200 |
| Waiting time | 3h | 5h |
| Pain | Mild | Severe |
| Frequency | Once every two years | Once every three years |
| Sensitivity | 65% | 95% |
| Your choice is? □ Programme 1 □ Programme 2 □ Choose neither | | |

1. which of these programmes would you prefer?

| Attribute | Programme 1 | Programme 2 |
| --- | --- | --- |
| Cost | 400 | 600 |
| Waiting time | 5h | 1h |
| Pain | None | Mild |
| Frequency | Once every two years | Once every three years |
| Sensitivity | 65% | 95% |
| Your choice is? □ Programme 1 □ Programme 2 □ Choose neither | | |

1. which of these programmes would you prefer?

| Attribute | Programme 1 | Programme 2 |
| --- | --- | --- |
| Cost | 400 | 600 |
| Waiting time | 5h | 1h |
| Pain | Mild | Severe |
| Frequency | Once a year | Once every two years |
| Sensitivity | 95% | 35% |
| Your choice is? □ Programme 1 □ Programme 2 □ Choose neither | | |

1. which of these programmes would you prefer?

| Attribute | Programme 1 | Programme 2 |
| --- | --- | --- |
| Cost | 200 | 400 |
| Waiting time | 1h | 3h |
| Pain | Severe | None |
| Frequency | Once every three years | Once every three years |
| Sensitivity | 65% | 95% |
| Your choice is? □ Programme 1 □ Programme 2 □ Choose neither | | |

1. which of these programmes would you prefer?

| Attribute | Programme 1 | Programme 2 |
| --- | --- | --- |
| Cost | 400 | 600 |
| Waiting time | 3h | 5h |
| Pain | None | Mild |
| Frequency | Once a year | Once every two years |
| Sensitivity | 95% | 35% |
| Your choice is? □ Programme 1 □ Programme 2 □ Choose neither | | |

6. which of these programmes would you prefer?

| Attribute | Programme 1 | Programme 2 |
| --- | --- | --- |
| Cost | 400 | 600 |
| Waiting time | 1h | 3h |
| Pain | Mild | Severe |
| Frequency | Once every three years | Once a year |
| Sensitivity | 35% | 65% |
| Your choice is? □ Programme 1 □ Programme 2 □ Choose neither | | |

7. which of these programmes would you prefer?

| Attribute | Programme 1 | Programme 2 |
| --- | --- | --- |
| Cost | 400 | 600 |
| Waiting time | 3h | 5h |
| Pain | Mild | Severe |
| Frequency | Once every three years | Once a year |
| Sensitivity | 35% | 65% |
| Your choice is? □ Programme 1 □ Programme 2 □ Choose neither | | |

8. which of these programmes would you prefer?

| Attribute | Programme 1 | Programme 2 |
| --- | --- | --- |
| Cost | 200 | 400 |
| Waiting time | 1h | 3h |
| Pain | None | Mild |
| Frequency | Once every two years | Once every three years |
| Sensitivity | 95% | 35% |
| Your choice is? □ Programme 1 □ Programme 2 □ Choose neither | | |

9. which of these programmes would you prefer?

| Attribute | Programme 1 | Programme 2 |
| --- | --- | --- |
| Cost | 600 | 200 |
| Waiting time | 1h | 3h |
| Pain | None | Mild |
| Frequency | Once a year | Once every two years |
| Sensitivity | 65% | 95% |
| Your choice is? □ Programme 1 □ Programme 2 □ Choose neither | | |

10. which of these programmes would you prefer?

| Attribute | Programme 1 | Programme 2 |
| --- | --- | --- |
| Cost | 400 | 600 |
| Waiting time | 5h | 1h |
| Pain | None | Mild |
| Frequency | Once every two years | Once every three years |
| Sensitivity | 65% | 95% |
| Your choice is? □ Programme 1 □ Programme 2 □ Choose neither | | |

Block 3

1. which of these programmes would you prefer?

| Attribute | Programme 1 | Programme 2 |
| --- | --- | --- |
| Cost | 200 | 400 |
| Waiting time | 5h | 1h |
| Pain | Severe | None |
| Frequency | Once every three years | Once a year |
| Sensitivity | 95% | 35% |
| Your choice is? □ Programme 1 □ Programme 2 □ Choose neither | | |

1. which of these programmes would you prefer?

| Attribute | Programme 1 | Programme 2 |
| --- | --- | --- |
| Cost | 400 | 600 |
| Waiting time | 1h | 3h |
| Pain | Severe | None |
| Frequency | Once every two years | Once every three years |
| Sensitivity | 65% | 95% |
| Your choice is? □ Programme 1 □ Programme 2 □ Choose neither | | |

1. which of these programmes would you prefer?

| Attribute | Programme 1 | Programme 2 |
| --- | --- | --- |
| Cost | 600 | 200 |
| Waiting time | 3h | 5h |
| Pain | Mild | Mild |
| Frequency | Once every three years | Once a year |
| Sensitivity | 95% | 35% |
| Your choice is? □ Programme 1 □ Programme 2 □ Choose neither | | |

1. which of these programmes would you prefer?

| Attribute | Programme 1 | Programme 2 |
| --- | --- | --- |
| Cost | 200 | 400 |
| Waiting time | 3h | 5h |
| Pain | Severe | None |
| Frequency | Once a year | Once every two years |
| Sensitivity | 35% | 65% |
| Your choice is? □ Programme 1 □ Programme 2 □ Choose neither | | |

1. which of these programmes would you prefer?

| Attribute | Programme 1 | Programme 2 |
| --- | --- | --- |
| Cost | 400 | 600 |
| Waiting time | 5h | 1h |
| Pain | None | Mild |
| Frequency | Once every three years | Once a year |
| Sensitivity | 65% | 95% |
| Your choice is? □ Programme 1 □ Programme 2 □ Choose neither | | |

6. which of these programmes would you prefer?

| Attribute | Programme 1 | Programme 2 |
| --- | --- | --- |
| Cost | 600 | 200 |
| Waiting time | 3h | 1h |
| Pain | Mild | Severe |
| Frequency | Once every two years | Once every three years |
| Sensitivity | 35% | 65% |
| Your choice is? □ Programme 1 □ Programme 2 □ Choose neither | | |

7. which of these programmes would you prefer?

| Attribute | Programme 1 | Programme 2 |
| --- | --- | --- |
| Cost | 600 | 200 |
| Waiting time | 5h | 1h |
| Pain | Severe | None |
| Frequency | Once a year | Once every two years |
| Sensitivity | 65% | 95% |
| Your choice is? □ Programme 1 □ Programme 2 □ Choose neither | | |

8. which of these programmes would you prefer?

| Attribute | Programme 1 | Programme 2 |
| --- | --- | --- |
| Cost | 200 | 400 |
| Waiting time | 5h | 1h |
| Pain | Mild | Severe |
| Frequency | Once a year | Once every two years |
| Sensitivity | 65% | 95% |
| Your choice is? □ Programme 1 □ Programme 2 □ Choose neither | | |

9. which of these programmes would you prefer?

| Attribute | Programme 1 | Programme 2 |
| --- | --- | --- |
| Cost | 400 | 600 |
| Waiting time | 1h | 3h |
| Pain | Severe | None |
| Frequency | Once every two years | Once every three years |
| Sensitivity | 95% | 95% |
| Your choice is? □ Programme 1 □ Programme 2 □ Choose neither | | |

10. which of these programmes would you prefer?

| Attribute | Programme 1 | Programme 2 |
| --- | --- | --- |
| Cost | 400 | 600 |
| Waiting time | 1h | 3h |
| Pain | Severe | None |
| Frequency | Once every two years | Once every three years |
| Sensitivity | 65% | 95% |
| Your choice is? □ Programme 1 □ Programme 2 □ Choose neither | | |

Block 4

1. which of these programmes would you prefer?

| Attribute | Programme 1 | Programme 2 |
| --- | --- | --- |
| Cost | 200 | 400 |
| Waiting time | 1h | 3h |
| Pain | None | Mild |
| Frequency | Once a year | Once every two years |
| Sensitivity | 35% | 65% |
| Your choice is? □ Programme 1 □ Programme 2 □ Choose neither | | |

1. which of these programmes would you prefer?

| Attribute | Programme 1 | Programme 2 |
| --- | --- | --- |
| Cost | 400 | 600 |
| Waiting time | 3h | 5h |
| Pain | Mild | Severe |
| Frequency | Once every two years | Once a year |
| Sensitivity | 65% | 95% |
| Your choice is? □ Programme 1 □ Programme 2 □ Choose neither | | |

1. which of these programmes would you prefer?

| Attribute | Programme 1 | Programme 2 |
| --- | --- | --- |
| Cost | 600 | 200 |
| Waiting time | 5h | 1h |
| Pain | Severe | None |
| Frequency | Once every three years | Once a year |
| Sensitivity | 95% | 35% |
| Your choice is? □ Programme 1 □ Programme 2 □ Choose neither | | |

1. which of these programmes would you prefer?

| Attribute | Programme 1 | Programme 2 |
| --- | --- | --- |
| Cost | 400 | 600 |
| Waiting time | 1h | 3h |
| Pain | None | Mild |
| Frequency | Once a year | Once every two years |
| Sensitivity | 35% | 65% |
| Your choice is? □ Programme 1 □ Programme 2 □ Choose neither | | |

1. which of these programmes would you prefer?

| Attribute | Programme 1 | Programme 2 |
| --- | --- | --- |
| Cost | 600 | 200 |
| Waiting time | 5h | 1h |
| Pain | None | Mild |
| Frequency | Once every two years | Once every three years |
| Sensitivity | 35% | 65% |
| Your choice is? □ Programme 1 □ Programme 2 □ Choose neither | | |

6. which of these programmes would you prefer?

| Attribute | Programme 1 | Programme 2 |
| --- | --- | --- |
| Cost | 200 | 400 |
| Waiting time | 1h | 5h |
| Pain | Mild | None |
| Frequency | Once a year | Once a year |
| Sensitivity | 65% | 95% |
| Your choice is? □ Programme 1 □ Programme 2 □ Choose neither | | |

7. which of these programmes would you prefer?

| Attribute | Programme 1 | Programme 2 |
| --- | --- | --- |
| Cost | 400 | 600 |
| Waiting time | 3h | 5h |
| Pain | Severe | None |
| Frequency | Once a year | Once every two years |
| Sensitivity | 95% | 95% |
| Your choice is? □ Programme 1 □ Programme 2 □ Choose neither | | |

8. which of these programmes would you prefer?

| Attribute | Programme 1 | Programme 2 |
| --- | --- | --- |
| Cost | 200 | 400 |
| Waiting time | 3h | 5h |
| Pain | Mild | Severe |
| Frequency | Once every two years | Once every three years |
| Sensitivity | 95% | 35% |
| Your choice is? □ Programme 1 □ Programme 2 □ Choose neither | | |

9. which of these programmes would you prefer?

| Attribute | Programme 1 | Programme 2 |
| --- | --- | --- |
| Cost | 600 | 200 |
| Waiting time | 3h | 5h |
| Pain | None | Mild |
| Frequency | Once every three years | Once a year |
| Sensitivity | 35% | 65% |
| Your choice is? □ Programme 1 □ Programme 2 □ Choose neither | | |

10. which of these programmes would you prefer?

| Attribute | Programme 1 | Programme 2 |
| --- | --- | --- |
| Cost | 400 | 600 |
| Waiting time | 3h | 5h |
| Pain | Mild | Severe |
| Frequency | Once every two years | Once a year |
| Sensitivity | 65% | 95% |
| Your choice is? □ Programme 1 □ Programme 2 □ Choose neither | | |
